# Chemosensory Perception of Predators by Larval Amphibians Depends on Water Quality

**DOI:** 10.1371/journal.pone.0131516

**Published:** 2015-06-26

**Authors:** Rachael R. Troyer, Andrew M. Turner

**Affiliations:** Department of Biology, Clarion University, Clarion, PA, United States of America; Gettysburg College, UNITED STATES

## Abstract

The acquisition of sensory information by animals is central to species interactions. In aquatic environments, most taxa use chemical cues to assess predation risk and other key ecological factors. A number of laboratory studies suggest that anthropogenic pollutants can disrupt chemoreception, even when at low, non-toxic concentrations, but there are few tests of whether real-world variation in water quality affects chemoreception. Here we investigate whether chemosensory perception of predators by the gray treefrog, *Hyla versicolor*, depends on water quality. We evaluated the anti-predator response of anuran tadpoles housed in water collected from three sites that represent strong contrasts in the concentration and types of dissolved solids: de-chlorinated tap water, water from an impaired stream, and treated wastewater effluent. Behavioral assays were conducted in laboratory aquaria. Chemical cues associated with predation were generated by feeding tadpoles to dragonfly predators held in containers, and then transferring aliquots of water from dragonfly containers to experimental aquaria. Tadpoles housed in tap water responded to predator cues with an activity reduction of 49%. Tadpoles housed in stream water and wastewater effluent responded to predator cues by reducing activity by 29% and 24% respectively. The results of factorial ANOVA support the hypothesis that the response to predator cues depended on water type. These results show that alteration of the chemical environment can mediate chemical perception of predators in aquatic ecosystems. Because most aquatic species rely on chemoreception to gather information on the location of food and predators, any impairment of sensory perception likely has important ecological consequences.

## Introduction

Sensory information plays a central but often unappreciated role in shaping species interactions. Foragers require accurate data regarding the location and identity of prey in order to successfully acquire food, and prey require data regarding the location, identity, and abundance of predators in order to successfully employ anti-predator defenses. The outcome of these important processes depends on the quality and quantity of sensory information [1]. Thus, species interactions emerge from information conveyed among individuals, and species embedded in an ecological community are connected by an “information web” just as they are interconnected by a food web [2].

In aquatic ecosystems, reception of chemical cues that provide information regarding predators, food, and potential mates is likely the predominant sensory modality [3–4]. The conveyance of these chemical cues, often referred to as infochemicals [5], plays an important role in the ecology of many aquatic communities. For example, by using odors in the water, the freshwater snail *Physa acuta* can assess the proximity of predators in its immediate environment, the history of predator presence, the species-level identity of the predators, and even discern the recent diet of potential predators [6–8]. Based on this detailed sensory information, *P*. *acuta* alters its behavior, morphology, and life-history in an adaptive manner. These shifts in phenotype, in turn, affect the snail’s resources, its competitors, and its predators [9]. In a similar manner, many larval anurans respond to predators with changes in activity, morphology, and life history, and these shifts have a strong influence on competition among anuran species [10–11]. In this way, species affect each other’s population growth rates not just by consuming and being consumed, but also by inducing shifts in the phenotypes of species with which they interact. Trait shifts that propagate through the food web and alter the strength of species interactions are called trait-mediated indirect effects. An accumulating body of evidence shows that trait-mediated indirect effects can play an important role in organizing communities [12]. Research also shows that for aquatic communities, chemical cues are often responsible for triggering trait-mediated indirect effects [1,13].

With growing awareness of the role played by chemical information in shaping species interactions has come a realization that alteration of the background “odorscape” can potentially disrupt chemical communication by altering or masking the chemical cues, and this disruption may cascade through the community [14–16]. A number of studies show that a wide variety of anthropogenic pollutants commonly found in surface waters can impair chemoreception [17–18], even when present at low, sublethal concentrations. Dissolved metals, surfactants, pesticides, nanoparticles, and pharmaceuticals have all been shown to impair chemoreception of aquatic organisms, and are termed “infodisruptors” [19]. However, most of these studies have presented animals with one focal disruptor at a time. Animals in their natural environment face the challenge of perceiving odors against a complex olfactory backdrop and are potentially faced with suites of infodisruptors acting in concert. They also have a greater opportunity in nature to acclimate to the prevailing olfactory climate. Thus, while we know from laboratory studies that animal chemoreception can be impaired, it is difficult to extend these results to the field, and we know less about the degree to which chemoreception is impaired in nature [1]. There is a need to broaden the approaches to studying infodisruption by incorporating elements of field environments into our studies.

Here we test whether variation in water quality affects the ability of larval amphibians to perceive predation risk and employ anti-predator defenses. We evaluated the anti-predator response of anuran tadpoles housed in water collected from three different sites that represent strong contrasts in the concentrations and types of dissolved solids. We hypothesized that the ability to perceive chemosensory cues and respond to predation risk would depend on water type.

## Methods

We tested whether the anti-predator response of gray treefrog tadpoles, *Hyla versicolor*, depends on water quality by conducting short-term experiments in which we manipulated perceived predation risk and water quality in a factorial design and assayed tadpole behavior. Gray treefrog tadpoles are adept at using chemical cues to assess predation risk and employ several defenses when confronted with predators, including the development of scarlet pigmentation in the tail, induction of deeper tails, and lower activity levels [20, 21]. These adaptive shifts in morphology and behavior have significant ecological consequences [22] and are mediated by the reception of chemical cues associated with predation [23]. Although gray treefrogs are primarily pond breeders, their behavioral plasticity and reliance on chemical cues makes them a good model system with which to investigate the effects of water quality on chemoreception.

Gray treefrog tadpoles were collected from outdoor water tanks, filled with a mixture of well water and rainwater, in which adult treefrogs had oviposited eggs that subsequently hatched and developed. Tadpoles were in mid-larval stage (approximately Gosner stage 25, 20–28mm total length) at time of collection, and no tadpoles were held more than four days between collection and use in an experiment. Tadpoles were housed in dechlorinated and aerated tap-water while held in the laboratory, and were fed to satiation with finely ground fish food. Predators in this study were late-instar larval dragonflies in the genus *Anax* (Aeschnidae), which are important consumers of hylid tadpoles [24]. Dragonflies (25–45mm in length) were collected from local ponds. Collections of both tadpoles and dragonflies were authorized by a permit granted by the Pennsylvania Fish and Boat Commission and remaining animals were returned to pond of origin at the conclusion of the study. Animal use protocols were approved by the Clarion University Institutional Animal Care and Use Committee, and all field activities were conducted on public lands and waters.

The experiment was conducted in a laboratory setting but used three different sources of water. Water types were chosen as to create a contrast of water sources with low, medium, and high total dissolved solids. Water sources were tap-water, water from a river moderately polluted by acid-mine drainage, and water drawn from the effluent of a wastewater treatment plant. It is certain that the concentration of other compounds, including particulate material, dissolved organics, and surfactants, also differed among the three water sources. We did not attempt to describe the chemical composition of the water or determine which compounds might have olfactory activity, but instead tested the more general hypothesis that differences in water composition, whatever they may be, will affect sensory perception.

For all three water sources, water was collected 24 hours before the experiment was initiated and maintained in aerated containers until added to experimental microcosms. At the time of each water collection, we measured pH and specific conductivity (Table 1). Tap water was chosen as a reference treatment based on the reasoning that the treatment process would yield water favorable to chemoreception. Our tap water was produced by the Clarion Borough municipal water treatment system (Clarion Country, Pennsylvania, USA), which supplies water drawn from the Clarion River and treated with activated charcoal before disinfection with chloramines. We used a sodium-thiosulfate based dechlorinator to neutralize the chloramine and circulated the water through an activated charcoal filter for 24 hours to further reduce residual odors and metals. The second source of water was Redbank Creek (Jefferson County, Pennsylvania). Redbank Creek drains a mostly forested watershed, but receives runoff from abandoned coal mines and has a relatively high concentration of dissolved metals, including iron and manganese. Segments of Redbank Creek adjacent to the study location are classified as “impaired” and are listed on the 303(d) list by the U.S. Environmental Protection Agency due to elevated concentrations of dissolved metals. The third water source was treated effluent from Brookville Wastewater Plant, which is discharged into Redbank Creek. The Brookville Wastewater plant is a modern high-performance treatment system that includes tertiary treatment and the disinfection/ dechlorination of effluent. Treated effluent was collected at the point that the discharge enters Redbank Creek.

**Table 1:** Basic water quality parameters for the three water sources used in this study.

Water Source	pH	Specific conductivity (μS/cm)
Tap water	7.63	210
River water	7.52	250
Treated wastewater	7.19	510

Water was collected on four dates, corresponding with four rounds of behavioral assays, and values shown here are mean values for the four water collection events. Specific conductivity is an index of total dissolved solids.

Behavioral assays were conducted in polyethylene washtubs (30 x 45 x 15 cm deep) filled with 7 L of water, drawn from the appropriate treatment. Five gray treefrog tadpoles were added to each washtub and allowed to acclimate 30 minutes before the initial behavioral assay was begun. We used tadpole activity level as our index of predator avoidance, as studies have shown that a reduction in activity is an adaptive response to dragonfly predators [25]. Activity was assayed both before treatment cues were added and after cue addition. Activity assays consisted of 20 second observations for each microcosm, with each microcosm observed in sequence. This observation cycle was repeated ten times for each observation bout, yielding 200 seconds of observation per microcosm for each bout. For each microcosm, the number of tadpoles active during the 20 second observation were counted, with any displacement movement scored as activity, and individual tadpoles were not scored more than once. The number of active tadpoles was divided by the total number of tadpoles (generally five) to yield the proportion of tadpoles that were active, and then the proportions were averaged across observations to yield an overall average activity level for a microcosm. Following the initial observation bout, predator cues were added, as described below, and post-cue observations began 30 minutes after cue addition. A total of 3 post-cue observation bouts were conducted, one 30 minutes after cue additions, one three hours after cue additions, and one 24 hours after cue additions. Preliminary analyses did not detect any effect of time after cue addition on activity, so we calculated an average activity per 20-second observation, based on all post-cue observations (which summed to ten minutes per microcosm), and used this value in all subsequent analyses. All observations were conducted by a single observer blind to treatment assignments.

Predator cues were generated by individually housing larval dragonflies in 12 drinking cups holding 80 ml of water each. Dragonfly cups were filled with water drawn from the appropriate water type treatment. Predators were placed in cups and fed one gray treefrog tadpole each 12 hours in advance of cue additions. Cues were transferred by removing the dragonfly from the cup and transferring the contents to the appropriate microcosm housing gray treefrog tadpoles.

Each behavioral assay consisted of 24 independent replicates (washtubs) randomly assigned to the six treatment combinations of predation risk and water type. Assays were repeated, using fresh animals and water collections, on four dates, yielding a total of 96 independent observations for the overall experiment. We analyzed the effects of predator cues and water type on activity level with a factorial analysis of variance. A significant predator cue x water type interaction term would provide support for the experimental hypothesis. Because four independent assays were conducted, and there were biological reasons to believe that treatment effects may change across dates, we entered assay date into the model as an additional factor. In addition, we used the pre-cue activity level as a covariate in the model. The omnibus ANOVA was followed with three focused one-way ANOVA’s, performed separately for each water type, testing whether cell means differed for predator and no predator treatments. Activity data were log transformed prior to analysis to promote homoscedasticity and meet the assumptions of ANOVA. Raw data are provided in S1 Table. In presenting results, we calculate the percent reduction in activity as ((% activity without predators)–(% activity with predators)) / (% activity without predators).

## Results

**Fig 1:**
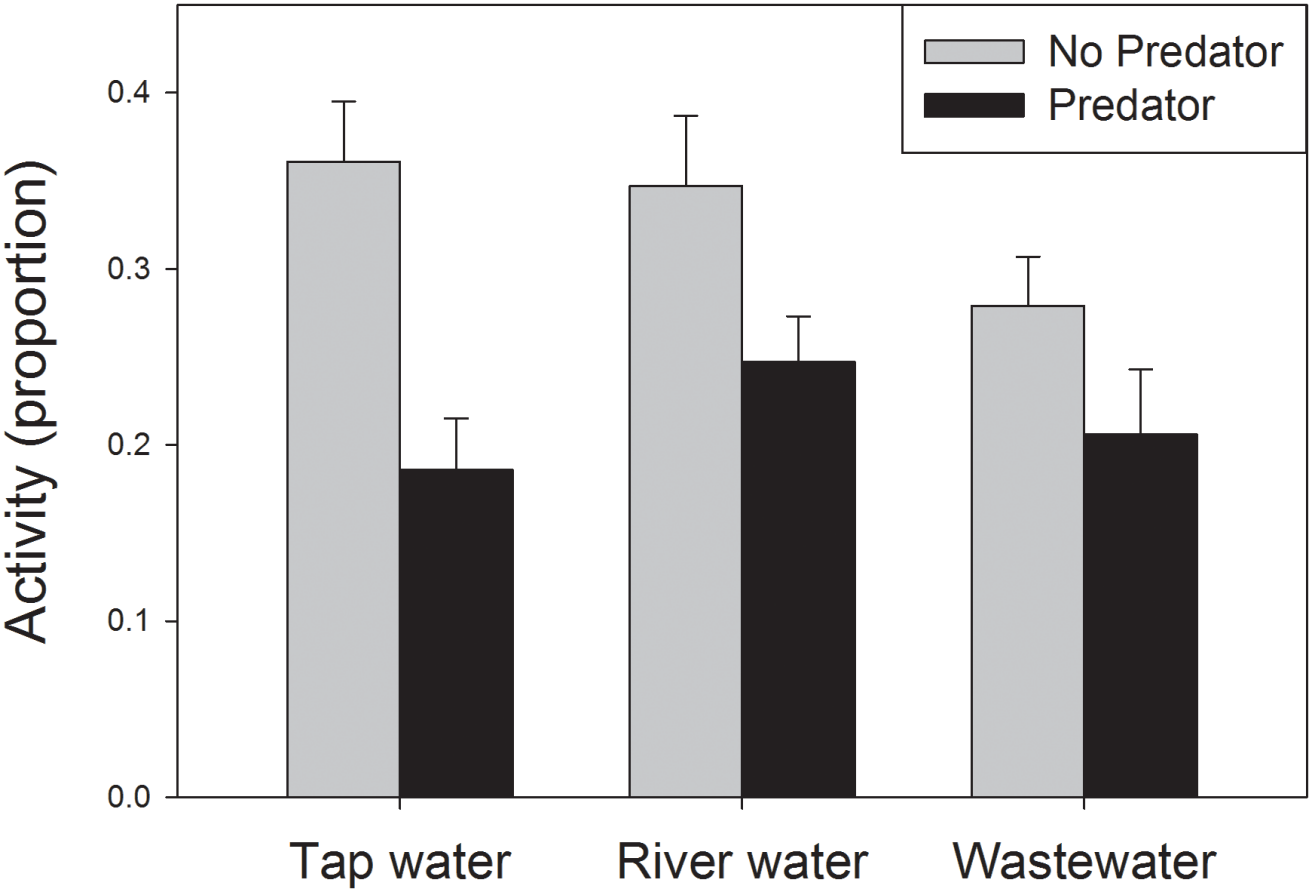
Effect of predator cues on activity of gray treefrog tadpoles. Tadpoles were housed in water drawn from three sources. Activity was scored as mean proportion of individuals that moved during 20-second observation bouts. Response to predator cues depended on water source. Bars represent one standard error, N = 16 replicates per treatment combination.

Dragonfly cues had a large effect on tadpole activity, as mean activity was reduced from 33% in the absence of predator cues to 21% in the presence of predator cues, a highly significant depression (Table 2). However, the effect of predator cues on tadpole activity depended on water treatment (Fig 1). Predator cues induced a 49% reduction in activity for tadpoles in tap water, a 29% reduction for tadpoles in river water, and a 24% reduction for tadpoles in wastewater effluent, yielding a significant interaction between the effects of predator cues and water type (Table 2). One-way ANOVA’s testing predator effects within each water type reveal that cues had highly significant effects on tadpoles housed in tap water (*F*_1,30_ = 17.3, *P* < 0.001), but predator cues had at best marginally significant effects on tadpoles housed in river water (*F*_1,30_ = 3.78, *P* = 0.06) and wastewater effluent (*F*_1,30_ = 4.20, *P* = 0.05).

**Table 2:** Analysis of variance testing overall effects of predator cues, water source, and date of assay on activity of gray treefrog tadpoles.

Source	df	Mean Square	*F*-ratio	*P*-value
Predator cues	1	0.338	22.718	< 0.001
Water source	2	0.044	2.979	0.057
Date of assay	3	0.038	2.541	0.063
Predator x Water	2	0.048	3.254	0.044
Water x Date	6	0.029	1.937	0.086
Predator x Date	3	0.009	0.626	0.601
Water x Predator x Date	6	0.004	0.279	0.945
Pre-cue activity level	1	0.584	39.325	<0.001
Error	71	0.015		

Overall model R^2^ = 0.63.

Water source, date of experimental trial, and the interaction of the two factors, each had small and only marginally significant effects on tadpole activity (Table 2). The remaining interaction terms were not significant (Table 2). Pre-cue activity level did account for a substantial portion of the overall variation in activity (Table 2), showing that variation among tubs within a treatment was consistent after cues were added. Survivorship of tadpoles housed in experimental microcosms for 24 hours averaged 94%, and was independent of water treatment (ANOVA: *P* > 0.20).

## Discussion

Our results show that real-world variation in water quality can have a significant effect on the ability of prey to perceive and respond appropriately to predation risk. Short-term exposure of tadpoles to a moderately polluted river or to treated wastewater effluent reduced the magnitude of the anti-predator response, presumably due to chemosensory impairment. We note, however, that tadpoles in both the river water and wastewater effluent treatments showed some tendency to lower activity in the presence of predators, demonstrating some robustness in their anti-predator response. We also note that in the field, wastewater effluent, which caused the highest degree of impairment, is diluted by river flow. In sum, our results support the hypothesis that sensory impairment occurs when animals are exposed to water collected from the field, but the degree of impairment we observed was moderate. Clearly, much additional research is needed before we can make any general statements regarding the extent of chemosensory impairment in nature.

We expected that sensory impairment would result in altered activity in the presence of predators, but inspection of Fig 1 shows that the significant interaction of predation risk and water quality was driven in part by a change in activity in the absence of predators (e.g. tap water versus waste water contrast), along with a lack of change in the presence of predators. It is difficult to predict or interpret the main effects on water type on baseline activity. More generally, we caution that treatment interactions like those shown in Fig 1 are subject to multiple interpretations, and that we lack a full mechanistic understanding of the processes that produced these patterns.

Our experiment was not designed to detect the identity of compounds responsible for sensory impairment. A review of the literature shows that a wide range of pollutants such as heavy metals, pesticides, nutrients, and surfactants can impair chemoreception in groups including fish, snails, crustaceans, and amphibians [15–17, 26–27]. However, because studies of infodisruption are heavily biased towards laboratory studies, we have little knowledge of the extent to which olfactory impairment actually occurs in the field. Studies that incorporate elements of a realistic ecological setting provide insight on the extent of chemosensory impairment in nature, but by necessity sacrifice some opportunity for a mechanistic understanding. In one example of such an approach, McPherson et al. [28] assayed behavior of wild fish in a field setting, and found that darters in lake polluted with dissolved copper did not exhibit the predator avoidance behaviors typical of fish in an unpolluted lake (see also [29, 30]). Such studies are unfortunately rare but complement mechanistic laboratory studies and provide a useful perspective on the potentially important role of chemosensory impairment in aquatic communities.

Our own study employed a hybrid lab-field approach, and it is possible that the short-term nature of the tadpole’s exposure to field-collected water influenced our results, as it precluded the opportunity for tadpoles to acclimate to the prevailing conditions. A short-term exposure to infodisruptors will tend to emphasize mechanisms associated with rapidly acting effects of water quality on infochemicals or on the olfactory receptors. Longer term experiments might result in significantly more pronounced effects if chronic exposure degrades sensory performance more than short-term exposure. However, it may result in weaker effects, if animals acclimate to the prevailing conditions. Ultimately, longer term and field based studies evaluating the perceptual ability of animals across water quality gradients are needed in order to resolve this important issue.

Our results have implications for the ongoing effort to evaluate the importance of trait-mediated indirect interactions in aquatic communities. Aquatic ecologists have in recent years expended a large effort to document the many strong effects of predation risk on prey behavior, morphology, life-history, and physiology. The general finding of this work is that traits of ecological importance are surprisingly plastic, and this result has been used to argue that trait-mediated indirect interactions are likely strong in nature [9, 13]. The vast majority of these studies have been conducted in the controlled setting of the laboratory or mesocosms, and there has been little attention paid to how variation in water quality or other potentially important chemical and physical constraints may affect olfaction [1, 31]. The results presented here show that the reaction to predators can be highly contingent on water quality. Experimentalists need to be aware of how the choice of water supply may affect experimental results, and they need to consider how real-world sensory constraints may affect species interactions when extending their results to the field (1, 24).

There are several potential mechanisms by which variation in water quality could affect chemoreception. Aquatic organisms are faced with the challenge of detecting small changes in the concentration of chemical cues associated with predators, food, or other relevant environmental features, even though these cues are embedded within a complex olfactory background [32]. Generally, infochemicals are a complex suite of proteins, amino acids, amines, and other soluble, low molecular weight compounds [32, 33]. The interfering compounds could bind directly with the infochemicals, changing the chemical structure [17]. Additionally, aquatic organisms have a wide variety of olfactory receptors that bind with a high degree of specificity to the infochemicals, and interfering compounds could block the binding process, or could induce conformational changes in chemosensory receptors, leading to their inactivity. For example, metals can effectively block ion channels at the receptor site, and can also be transported along nerves into more central portions of the nervous system and exert disruptive effects [34]. Finally, changes in the olfactory background can impair chemoreception simply by lowering the signal to noise ratio if the background compounds mask or substitute for the infochemicals [19].

The effects of sensory impairment on population, community, and ecosystem level processes are nearly unexplored, but may be quite important, as most aquatic organisms rely on chemoreception to cope with a complex and changing environment [35]. Constraints on information transfer may result in substantial shifts in community organization, highlighting the subtle ways in which sublethal doses of compounds may play a significant role in community ecology. For example, chemosensory disruption could increase or decrease prey survivorship, depending on whether predator or prey suffers the greater sensory impairment [36]. In the case of dragonfly naiads and tadpoles, dragonflies are largely visual predators, so the likely effect of chemosensory disruption will be to increase tadpole mortality rate. However, impairment of chemoreception could depress predator feeding rates and enhance survivorship of prey in systems with predators that rely heavily on chemosensory cues.

Our results have important applied implications. Human activities result in elevated loadings of many compounds into our surface waters, many of which are olfactory disruptors, including heavy metals, pesticides, plasticizers, and surfactants. If sensory disruption is as prevalent in our surface waters as the emerging evidence suggests, then it likely merits attention from regulators responsible for water quality. As is the case with the growing concern with endocrine disruptors and other emerging contaminants, pollutants that do not cause acute mortality can nevertheless disrupt population, community, and ecosystem level processes. Aquatic systems are at special risk both because of the prevalence of chemosensory perception and the widespread disruption of the chemical environment of our surface waters. Efforts to establish ecologically relevant thresholds for pollutants of any sort should consider not only the short-term effects on survival and reproduction of individuals, but also longer-term consequences that account for perceptual impairment.

## Supporting Information

S1 Table Raw data yielded by experiments. (PDF)
