# Supplementary material for: Chemosensory Perception of Predators by Larval Amphibians Depends on Water Quality
Source: PLoS One. 2015 Jun 26;10(6):e0131516. doi: 10.1371/journal.pone.0131516 (PMC4482611; doi:10.1371/journal.pone.0131516)
Supplement: S1 Table — (PDF) [file pone.0131516.s001.pdf]

S1 Table. Raw data yielded by experiments. For water source, wastew = treated wastewater effluent, river = river water, and tapw = tap water. For predator, no = tapwater control cues, and yes = dragonfly cues. Each assay involved 24 independent wash tubs, and the assay was repeated on four dates, as shown. Act_before = activity before cue addition (mean # moving per 20 seconds), averaged across 200 seconds of observation. Act_after = activity after cue addition (mean # moving per 20 seconds), averaged across 10 minutes of observation. T_before = log (x + 0.1) transformed activity before, and T_after = log (x + 0.1) transformed activity after.

**Water Source**	**Predator**	**Date**	**Act_before**	**Act_after**	**T_before**	**T_after**
wastew	No	10-Jun	0.5250	0.2917	-0.2041	-0.4071
wastew	No	10-Jun	0.6000	0.5667	-0.1549	-0.1761
wastew	No	10-Jun	0.2000	0.2667	-0.5229	-0.4357
wastew	No	10-Jun	0.2000	0.2000	-0.5229	-0.5229
wastew	Yes	10-Jun	0.2400	0.2600	-0.4685	-0.4437
wastew	Yes	10-Jun	0.0200	0.0267	-0.9208	-0.8973
wastew	Yes	10-Jun	0.4000	0.1750	-0.3010	-0.5607
wastew	Yes	10-Jun	0.2500	0.1417	-0.4559	-0.6168
river	No	10-Jun	0.2250	0.2083	-0.4881	-0.5110
river	No	10-Jun	0.2667	0.6000	-0.4357	-0.1549
river	No	10-Jun	0.4800	0.6533	-0.2366	-0.1230
river	No	10-Jun	0.1750	0.2750	-0.5607	-0.4260
river	Yes	10-Jun	0.2750	0.2333	-0.4260	-0.4771
river	Yes	10-Jun	0.3000	0.3167	-0.3979	-0.3802
river	Yes	10-Jun	0.2250	0.2167	-0.4881	-0.4994
river	Yes	10-Jun	0.1750	0.3583	-0.5607	-0.3388
tapw	No	10-Jun	0.2333	0.2333	-0.4771	-0.4771
tapw	No	10-Jun	0.0800	0.2400	-0.7447	-0.4685
tapw	No	10-Jun	0.4750	0.5417	-0.2403	-0.1927
tapw	No	10-Jun	0.3000	0.3600	-0.3979	-0.3372
tapw	Yes	10-Jun	0.2800	0.0333	-0.4202	-0.8751
tapw	Yes	10-Jun	0.1750	0.0917	-0.5607	-0.7175
tapw	Yes	10-Jun	0.1000	0.1083	-0.6990	-0.6812
tapw	Yes	10-Jun	0.1000	0.1667	-0.6990	-0.5740
wastew	No	15-Jun	0.0000	0.1867	-1.0000	-0.5426
wastew	No	15-Jun	0.1000	0.1800	-0.6990	-0.5528
wastew	No	15-Jun	0.0600	0.2533	-0.7959	-0.4518
wastew	No	15-Jun	0.2600	0.4467	-0.4437	-0.2623
wastew	Yes	15-Jun	0.0200	0.0733	-0.9208	-0.7611
wastew	Yes	15-Jun	0.0750	0.2833	-0.7570	-0.4164
wastew	Yes	15-Jun	0.0200	0.2333	-0.9208	-0.4771
wastew	Yes	15-Jun	0.2800	0.3467	-0.4202	-0.3500
river	No	15-Jun	0.1000	0.3133	-0.6990	-0.3837

river	No	15-Jun	0.0400	0.0933	-0.8539	-0.7137
river	No	15-Jun	0.0400	0.2467	-0.8539	-0.4601
river	No	15-Jun	0.3000	0.3667	-0.3979	-0.3310
river	Yes	15-Jun	0.0000	0.1167	-1.0000	-0.6642
river	Yes	15-Jun	0.1000	0.1333	-0.6990	-0.6320
river	Yes	15-Jun	0.1000	0.1667	-0.6990	-0.5740
river	Yes	15-Jun	0.0200	0.1133	-0.9208	-0.6709
tapw	No	15-Jun	0.1400	0.5067	-0.6198	-0.2170
tapw	No	15-Jun	0.1000	0.1867	-0.6990	-0.5426
tapw	No	15-Jun	0.2600	0.4667	-0.4437	-0.2467
tapw	No	15-Jun	0.2750	0.3750	-0.4260	-0.3233
tapw	Yes	15-Jun	0.1400	0.0733	-0.6198	-0.7611
tapw	Yes	15-Jun	0.2200	0.2200	-0.4949	-0.4949
tapw	Yes	15-Jun	0.1800	0.1333	-0.5528	-0.6320
tapw	Yes	15-Jun	0.3400	0.3467	-0.3565	-0.3500
wastew	No	23-Jun	0.2250	0.2667	-0.4881	-0.4357
wastew	No	23-Jun	0.3000	0.3667	-0.3979	-0.3310
wastew	No	23-Jun	0.3600	0.2133	-0.3372	-0.5040
wastew	No	23-Jun	0.1800	0.2667	-0.5528	-0.4357
wastew	Yes	23-Jun	0.0400	0.1200	-0.8539	-0.6576
wastew	Yes	23-Jun	0.1200	0.1933	-0.6576	-0.5326
wastew	Yes	23-Jun	0.3200	0.2800	-0.3768	-0.4202
wastew	Yes	23-Jun	0.1200	0.1467	-0.6576	-0.6079
river	No	23-Jun	0.0800	0.2267	-0.7447	-0.4859
river	No	23-Jun	0.0800	0.3000	-0.7447	-0.3979
river	No	23-Jun	0.1000	0.1417	-0.6990	-0.6168
river	No	23-Jun	0.2800	0.3267	-0.4202	-0.3699
river	Yes	23-Jun	0.1600	0.4667	-0.5850	-0.2467
river	Yes	23-Jun	0.0750	0.1417	-0.7570	-0.6168
river	Yes	23-Jun	0.3200	0.1467	-0.3768	-0.6079
river	Yes	23-Jun	0.1250	0.2917	-0.6478	-0.4071
tapw	No	23-Jun	0.1200	0.2733	-0.6576	-0.4279
tapw	No	23-Jun	0.4400	0.5133	-0.2676	-0.2123
tapw	No	23-Jun	0.1800	0.2733	-0.5528	-0.4279
tapw	No	23-Jun	0.2000	0.2467	-0.5229	-0.4601
tapw	Yes	23-Jun	0.1200	0.0467	-0.6576	-0.8337
tapw	Yes	23-Jun	0.2500	0.1250	-0.4559	-0.6478
tapw	Yes	23-Jun	0.2200	0.1933	-0.4949	-0.5326
tapw	Yes	23-Jun	0.1000	0.2400	-0.6990	-0.4685
wastew	No	25-Jun	0.1200	0.1000	-0.6576	-0.6990
wastew	No	25-Jun	0.1400	0.2267	-0.6198	-0.4859
wastew	No	25-Jun	0.1200	0.3333	-0.6576	-0.3632
wastew	No	25-Jun	0.2800	0.3000	-0.4202	-0.3979
wastew	Yes	25-Jun	0.0800	0.0533	-0.7447	-0.8144
wastew	Yes	25-Jun	0.0800	0.0533	-0.7447	-0.8144
wastew	Yes	25-Jun	0.5400	0.6200	-0.1938	-0.1427
wastew	Yes	25-Jun	0.3800	0.2867	-0.3188	-0.4127

river	No	25-Jun	0.3200	0.4267	-0.3768	-0.2785
river	No	25-Jun	0.3600	0.3400	-0.3372	-0.3565
river	No	25-Jun	0.3800	0.5733	-0.3188	-0.1718
river	No	25-Jun	0.2400	0.4667	-0.4685	-0.2467
river	Yes	25-Jun	0.1800	0.2933	-0.5528	-0.4052
river	Yes	25-Jun	0.2200	0.3600	-0.4949	-0.3372
river	Yes	25-Jun	0.2800	0.2867	-0.4202	-0.4127
river	Yes	25-Jun	0.2000	0.3067	-0.5229	-0.3908
tapw	No	25-Jun	0.1400	0.2200	-0.6198	-0.4949
tapw	No	25-Jun	0.2000	0.4000	-0.5229	-0.3010
tapw	No	25-Jun	0.3400	0.6400	-0.3565	-0.1308
tapw	No	25-Jun	0.0800	0.3067	-0.7447	-0.3908
tapw	Yes	25-Jun	0.2400	0.1733	-0.4685	-0.5633
tapw	Yes	25-Jun	0.1400	0.2800	-0.6198	-0.4202
tapw	Yes	25-Jun	0.4000	0.2800	-0.3010	-0.4202
tapw	Yes	25-Jun	0.1600	0.4600	-0.5850	-0.2518
